# Personality and Motives for Social Media Use When Physically Distanced: A Uses and Gratifications Approach

**DOI:** 10.3389/fpsyg.2021.607948

**Published:** 2021-06-14

**Authors:** Thomas Bowden-Green, Joanne Hinds, Adam Joinson

**Affiliations:** Information, Decisions and Operations Division, School of Management, University of Bath, Bath, United Kingdom

**Keywords:** Big Five, personality, motives, social media, uses and gratifications

## Abstract

This paper explores individuals’ motives for using social media when living under ‘social distancing’ conditions imposed during the COVID-19 pandemic, where they were instructed to physically distance from other people. Adopting a ‘uses and gratifications’ approach, and using a previously established five-factor scale, we examine the relationship between individuals’ motives for using social media and their personality traits. Hundred and eighty-nine social media users living in the United Kingdom completed surveys assessing their motives for using social media and their personality. Our findings demonstrate that participants were generally motivated to use social media to ‘pass time’ and to ‘maintain relationships.’ Further, we find that those high in extraversion in particular use social media to ‘maintain relationships.’ By comparing our findings to previous studies where face-to-face interaction was not restricted, our findings indicate that individuals’ motives for using social media change when they are placed under physical distancing restrictions. We reflect on the potential application of our findings for others experiencing similar conditions, such as those working in remote locations, as well as the potential implications for living in a post-pandemic world with increased virtual ‘meetings’ using social media.

## Introduction

In early 2020 the declaration of the COVID-19 pandemic let to unprecedented disruption to human interaction throughout the world. COVID-19 is a disease causing respiratory illness, resulting from infection through a novel coronavirus (World Health Organization, 2020). By July 2020 the pandemic had infected nearly 12 million people, and contributed to more than 500,000 deaths (Gutiérrez and Clarke, 2020). In response, many countries introduced laws and guidelines to restrict human interaction, referred to as ‘social distancing’ (United Kingdom [UK] Government, 2020a), intended to limit the spread of the virus. Although ‘social distancing’ is the term used by the United Kingdom government, the intention was to encourage individuals to ‘physically distance’ from each other, by restricting who they meet up with and by maintaining a 2-m distance from others in public spaces (United Kingdom [UK] Government, 2020b). In the United Kingdom, this led to a nationwide ‘lockdown’ requiring the population to remain at home where possible (United Kingdom [UK] Government, 2020c). This deliberate effort to restrict face-to-face contact to vital functions, such as performing ‘key work’ (i.e., those providing essential services such as nurses, teachers, police officers etc.) and shopping for basic necessities, inevitably applied significant limits on communication. Specifically, although many employers and educational institutions moved their activities to online environments such as Microsoft Teams and Zoom, many people also chose to communicate with one another socially during this ‘lockdown’ period using online communication channels in place of physical social engagement.

A wide range of evidence suggests that social media use increased following the physical distancing measures mandated in response to COVID-19. This included increased daily and monthly active use of Facebook (Facebook, 2020), a doubling of visitors to TikTok within the United Kingdom (Ofcom, 2020a) and more than doubling of weekly video calling using platforms such as WhatsApp, Facebook messenger, and Instagram (Ofcom, 2020a). Yet, although this evidence suggests that the increases in social media use may be a consequence of the pandemic, the numbers themselves do not explain why. So, why would the conditions of a pandemic motivate individuals to increase their use of social media? This paper therefore explores the motives for social media use under physically distanced conditions, paying specific attention to the relationship between motives and personality traits.

Previous studies have demonstrated that motives for using social media platforms can be driven by a range of desired ‘uses’ or ‘gratifications’ (such as Joinson, 2008). However, as well as differences in motivation, social media usage also varies according to individual characteristics such as demography (see Gil-Clavel and Zagheni, 2019) and personality traits (such as Bowden-Green et al., 2020). Indeed, these individual characteristics have been shown to affect motivation (Hollenbaugh and Ferris, 2014), thus in turn leading to the observed differences in usage.

Uses and gratifications theory developed as a means of understanding individuals’ motivations to receive communication via a given medium. A uses and gratifications approach acknowledges that an audience actively chooses the media it receives (Katz et al., 1974), linking a need for gratification with the choice of a specific medium that will satisfy the need. This assumes that people are ‘sufficiently self-aware’ to accurately report their ‘interests and motives’ (Katz et al., 1974). Katz et al. (1974) outlined ‘social’ factors that might create a need for media use. Others focus on satisfaction of individual needs (McQuail et al., 1972; Rosengren, 1974), such as ‘belonging,’ ‘esteem,’ and ‘self-actualization.’

Following the establishment of uses and gratifications theory, the approach has been applied to a variety of media types (see Ruggiero, 2000 for a review). Among the well-cited contributions, Katz et al. (1973) demonstrated that specific mass media satisfy specific social and psychological needs, for example identifying that “books” satisfy a need “to escape from the reality of everyday life.” Although most early research focused on ‘traditional’ broadcast and print media, Perse and Courtright (1993) later added computers to the potential media for which uses and gratifications were identified. As the media landscape has evolved, recent work has focused specifically on a range of social media including Facebook Groups (Park et al., 2009), Everything2.com (Lampe et al., 2010), and Qzone (Apaolaza et al., 2014). Yet, as Facebook has grown to become the world’s largest social media platform (Statista, 2020), most recent research has focused on understanding motives for Facebook use. Early research identified the desire to find new and old friends (Raacke and Bonds-Raacke, 2008) on both Facebook and MySpace, although a range of specific uses and gratifications factors have since been developed for researching social media motivation (such as Joinson, 2008; Sundar and Limperos, 2013). As well as a general motivation for Facebook users to ‘pass time’ and seek ‘relaxing entertainment’ (Papacharissi and Mendelson, 2011), findings for Facebook to date also include ‘content gratification’ as a motive for those spending a long duration online (Joinson, 2008), and a need for ‘social connection’ among high-frequency users (Joinson, 2008). Excessive users of social networks, however, are driven by diversion, self-presentation, and relationship building (Chen and Kim, 2013).

Specific functions within each platform are also linked with specific motives, such as commenting relating to a desire to socially interact and to seek relaxing entertainment (Smock et al., 2011), and status updates being driven by a desire to share information (Smock et al., 2011), form relationships, and maintain relationships (Yang and Brown, 2013).

In contrast to the extensive body of work on uses and gratifications generally, research that compares social media uses and gratifications with personality traits is limited. Results to date suggest that individual personality characteristics alter the motives for using social media as users seek different benefits, such as those higher in agreeableness seeking a ‘Virtual Community,’ and those higher in Openness seeking ‘Relationship Maintenance’ (Ferris and Hollenbaugh, 2018). In the context of physically distanced situations, the current study therefore seeks to understand how personality traits affect the desire to use social media, given that many needs, such as social interaction, cannot be fulfilled offline.

The identification of personality traits according to the five-factor model is based on initial analysis of language used to describe people. One of the first pieces of research studied all 18,000 personality descriptors in the English dictionary, identifying 4,500 of these as ‘personality’ (Allport and Odbert, 1936). Using cluster analyses and factor analysis, 4,500 traits were reduced to just 35 variables, contributing to five ‘factors (Fiske, 1949): Extraversion (or introversion), agreeableness (or antagonism), conscientiousness (or lack of direction), neuroticism (or emotional stability), and openness to experience (versus closeness). These are now known as the ‘Big Five’ (Goldberg, 1981).

McCrae and Costa (2003) recognized that within these five ‘factors’ are many more behavioral ‘facets.’ For example, expressing feelings (such as excitement) is a behavioral facet of ‘openness to experience.’ Various multi-item questionnaires have been suggested to test the presence of each facet and then score participants against these five overall factors. These include the 44-item Big Five Inventory [BFI] (John et al., 1991), 50-item questionnaire (with 10 bi-polar adjective scales per factor) (Goldberg, 1992), 60-item neuroticism, extraversion, openness five factor inventory [NEO-FFI] (Costa and McCrae, 1992), 100-item questionnaire (unipolar) (Goldberg, 1992), and the 240-item neuroticism, extraversion, openness [NEO] personality inventory (Costa and McCrae, 1992).

Using a variety of personality questionnaires (through which respondents self-report their personality), there is lots of evidence to suggest that behavior on social media is linked to personality traits (Ong et al., 2017). For example, a large body of research has discovered relationships between personality traits and ‘posting’ content (Ong et al., 2011; Bachrach et al., 2012; Wang et al., 2012; Lee et al., 2014; Shen et al., 2015; Cheevasuntorn et al., 2017; Yoong et al., 2017; Casado-Riera and Carbonell, 2018; Mo et al., 2018; Seidman, 2019), ‘liking’ others’ content (Bachrach et al., 2012; Lee et al., 2014; Marshall et al., 2015; Shchebetenko, 2019), and ‘commenting’ (Gosling et al., 2011; Lee et al., 2014; Marshall et al., 2015; Wang et al., 2018). There is therefore a large body of evidence to suggest that relationships exist between traits and social media behavior. Specific trends in the findings include people with high trait neuroticism posting longer updates (such as Bai et al., 2012) and negative emotions (such as Kern et al., 2014); whereas those high in extraversion tend to use positive words (such as Hall et al., 2014), use social media frequently (such as Correa et al., 2010) and regularly post content (such as Bachrach et al., 2012). For reviews of these findings, see Bowden-Green et al. (2020, 2021).

Reflecting previous offline studies linking traits with motives for offline media use (such as Finn, 1997; Krcmar and Kean, 2005), Hollenbaugh and Ferris (2014) then sought to understand people’s motives for using social media. Among the relationships identified in research to date, extraversion has been linked with the motive to connect with new friends (Bhattacharya et al., 2014; Orchard et al., 2014) and existing peers (Bhattacharya et al., 2014), and to share information about themselves (Mishra and Ayatham, 2017). Krishnan and Atkin (2014) also demonstrated that, for those high in extraversion, infotainment was likely to be a motive for using social networks. Although people with high trait neuroticism tend to have small social networks, social interaction is a motivation for individuals to use social media (Hughes et al., 2012). Neuroticism also relates to social media use as a means of escapism (Orchard et al., 2014) or as a means of coping with pressure (Marino et al., 2016), with social media satisfying a desire for acceptance and inclusion (Marshall et al., 2015). Openness has been related to use of social media to connect with like-minded people and find new friends (Bhattacharya et al., 2014), use social networking sites for learning (Chou and Chiu (2015) and sharing information Marshall et al., 2015), and to search for products (Bhattacharya et al., 2014; Mishra and Ayatham, 2017). Those high in agreeableness are also likely to use social media to maintain relationships (Horzum, 2016) and to use Pinterest specifically to entertain and inform themselves (Lin et al., 2017). Conscientiousness has been associated with a motive to connect with peers (Bhattacharya et al., 2014) and maintain relationships (Horzum, 2016).

Yet, although motives for social media use were identified in a recent study (Ferris and Hollenbaugh, 2018) for agreeableness (joining a ‘Virtual Community’), neuroticism (‘Companionship’), and openness (‘Companionship,’ ‘Exhibitionism,’ and ‘Relationship Maintenance’), neither this study nor a previous study using the same ‘uses and gratifications’ scale (Hollenbaugh and Ferris, 2014) identified a significant motive for use of social media by those high in extraversion. This is despite other measures suggesting that people high in extraversion need to ‘connect’ (Bhattacharya et al., 2014; Orchard et al., 2014; Scherr and Brunet, 2017), communicate (Marshall et al., 2015; Horzum, 2016), and socially interact on social media (Eşkisu et al., 2017; Lin et al., 2017).

Although there is no direct comparison to conditions during a pandemic, previous studies have related Big Five personality traits to other situations of social isolation. Findings include increased feelings of loneliness for those scoring higher for neuroticism (Wang and Dong, 2018), but decreased feelings of loneliness for those scoring higher for conscientiousness (Wang and Dong, 2018; Buecker et al., 2020), extraversion and agreeableness (Buecker et al., 2020). Further, introverts have been found to perform well cognitively in isolated environments such as the Antarctic winter (Rosnet et al., 2000), when tested against a range of measures including memory, grammatical reasoning, and reaction time. These limited findings suggest that in situations of enforced physical distancing, introverts may feel comfortable when face-to-face interaction is restricted, whereas those scoring highly for neuroticism may not.

Studies of social media use during the current COVID-19 pandemic are understandably limited in number so far, however, these limited findings have tended to focus on the dissemination of information (Depoux et al., 2020; Islam et al., 2020; Rosenberg et al., 2020; Naeem, 2021), social media discourse (Chen et al., 2020; Koh and Liew, 2020), and mental health, such as anxiety (Ahmad and Murad, 2020; Wheaton et al., 2020; Zhao and Zhou, 2020). We have identified very few studies on specific social media use patterns (Drouin et al., 2020). We believe our study will be the first during the pandemic to focus on motives, with particular comparison to pre-pandemic conditions.

In this exploratory study, we examine whether previously established relationships between motives and individual characteristics, such as personality traits, have altered under physically distanced conditions. This study seeks to explore whether the evidence of increased social media use (Facebook, 2020; Ofcom, 2020a) is driven by specific motives and in turn by specific personality traits. Based on these findings, we therefore seek to answer the following questions:

**RQ1: What are people’s motives for using social media when instructed to physically distance?**

**RQ2: Does personality predict motives for using social media when instructed to physically distance?**

**RQ3: How do personality traits relate to use of specific social media platforms when instructed to physically distance?**

## Materials and Methods

### Participants

Participants were recruited through the Prolific online data collection tool^1^ and received £2 payment for their completion of the questionnaire. Prolific is an online participant recruitment tool, enabling researchers to recruit carefully screened participants for online research. Participants who have already signed up to participate in online research are invited to take part in specific studies via email if they meet the demographic requirements of the researcher. A total of 218 responses were received. Twenty participants did not fully complete the questionnaire. A preliminary question assessed social media use to ensure that participants were social media users; nine participants were removed as they did not use any of the top ten social media platforms. After also removing incomplete responses, the sample size was 189.

All participants were adults aged between 18 and 75 years (*M* = 36, *SD* = 13.6), who were living in the United Kingdom at the time of the study. Testing for skewness revealed a score of 0.83, indicating that more of our participants were at the lower end of our age range. Hundred and sixteen (57%) were male and 82 (43%) female.

### Procedure

#### Survey

Participants completed an online questionnaire created in Qualtrics that took around 20 min to complete. Data were collected on 18th and 19th May 2020 during the first national ‘lockdown’ in the United Kingdom. The survey comprised a personality test plus a series of questions regarding their motives for social media use since 23rd March 2020 when physical distancing measures were first instructed by the United Kingdom government (United Kingdom [UK] Government, 2020b). All participants gave consent for this data to be used for research purposes, and they were informed that they were free to withdraw at any time. All data collected were anonymous. Ethical approval was granted by the School of Management’s Ethics Committee at the University of Bath prior to undertaking this research.

#### Instruments

The online questionnaire measured motives for using social media using the 24-item scale created by Hollenbaugh and Ferris (2014). As described by Hollenbaugh and Ferris (2014), this scale is the result of factor analysis on 39 items, originally comprising items from Sheldon (2008), Barker and Ota (2011), and Hollenbaugh (2011). The resulting five factors included measurement of ‘Virtual Community’ with seven items assessing the use of social media to forge new relationships, ‘Companionship’ with five items assessing social media use to compensate for loneliness, ‘Exhibitionism’ with five items assessing the use of social media to get attention, ‘Relationship Maintenance’ using five items to assess social media use to sustain existing relationships, and ‘Passing Time’ using two items to assess the motive to relieve boredom through social media use.

Participants responded to 24 randomized statements using a Likert scale (*Strongly disagree, disagree, neither agree nor disagree, agree, strongly agree*). The answers then contributed to five previously established factors (Hollenbaugh and Ferris, 2014): participating in a ‘Virtual Community,’ seeking ‘Companionship,’ ‘Exhibitionism,’ ‘Relationship Maintenance,’ and ‘Passing Time.’ According to Ferris and Hollenbaugh (2018) the scale has good internal consistency, with a Cronbach’s alpha coefficient reported of α = 0.89 for ‘Virtual Community,’ α = 0.94 for ‘Companionship,’ α = 0.90 for ‘Exhibitionism,’ α = 0.87 for ‘Relationship Maintenance,’ and *r* = 0.66 for ‘Passing Time’ (two items). In the current study, the overall Cronbach’s alpha coefficient was α = 0.88 for ‘Virtual Community,’ α = 0.91 for ‘Companionship,’ α = 0.84 for ‘Exhibitionism,’ α = 0.81 for ‘Relationship Maintenance,’ and *r* = 0.70 for ‘Passing Time’ (two items). Although the Hollenbaugh and Ferris (2014) scale was originally used to assess motives for using Facebook specifically, the current study sought to understand motives for a range of social media; therefore, the word ‘Facebook’ was replaced in the questions with the broader words ‘social media.’ Participants were asked to signal the extent to which they agreed with each of the 24 statements describing their motives for social media use since 23rd March 2020.

In order to relate these motives to use of specific social media, duration data was collected to provide an objective measure of social media use. Apple introduced a feature called ‘screen time’ for iPhones running version 9 of its operating software onward. This enables users to report their weekly time spent on each app, for specific functions in general, and on the phone as a whole. Therefore, those participants who use Apple iPhones as their primary device for social purposes were also asked to report data for use of each of the ten largest social media platforms by user number in the UK based on industry data produced by GlobalWebIndex (reported by We Are Social, 2020). As Apple iPhone ownership is not universal, this data was collected for a subset (*n* = 78) of the participants.

Personality traits were measured using the 120-item IPIP-NEO-120 scale provided by Johnson (2014). Participants responded to 120 randomized statements using a Likert scale (*Very inaccurate, moderately inaccurate, neither accurate nor inaccurate, moderately accurate, very accurate*). After reversing scores for negatively worded statements, the 120-items then give scores for 30 ‘facets’ which contribute to the ‘Big Five trait’ factors (neuroticism, extraversion, openness, agreeableness, and conscientiousness). According to Johnson (2014), the scale has a reported Cronbach’s alpha coefficient of α = 0.88 for neuroticism, α = 0.84 for extraversion, α = 0.85 for openness, α = 0.81 for agreeableness, and α = 0.84 for conscientiousness. In the current study, the Cronbach’s alpha coefficient was α = 0.92 for neuroticism, α = 0.91 for extraversion, α = 0.79 for openness, α = 0.86 for agreeableness, and α = 0.88 for conscientiousness.

## Results

Following the order of our research questions, we firstly considered the overall motives for using social media in a physically distanced situation. We then looked at variations in the motives for using social media according to personality traits, reporting in particular on where personality predicted particular motives. Lastly, we also present our findings for the platforms used within this overall ‘social media use,’ according to duration data. Table 1 provides descriptive statistics of all variables in this study.

**TABLE 1:** Means, standard deviations, skewness and kurtosis for all study variables.

	*N*	Minimum	Maximum	Mean	*SD*	Skewness		Kurtosis	
		
						Statistic	*SE*	Statistic	*SE*
Age	189	18	75	36.33	13.61	0.83	0.177	–0.07	0.35
Neuroticism	189	29	119	73.82	17.53	–0.09	0.177	–0.18	0.35
Extraversion	189	26	108	70.78	15.55	–0.24	0.177	0.01	0.35
Openness	189	47	112	79.90	11.18	0.06	0.177	–0.26	0.35
Agreeableness	189	55	118	90.66	11.89	–0.30	0.177	–0.29	0.35
Conscientiousness	189	49	117	84.88	13.42	–0.18	0.177	–0.29	0.35
***Social media motives***									
Virtual community	189	1.00	4.67	1.91	0.87	1.00	0.177	0.52	0.35
Companionship	189	1.00	5.00	2.53	1.10	0.18	0.177	–1.02	0.35
Exhibitionism	189	1.00	3.80	1.72	0.74	0.89	0.177	–0.19	0.35
Relationship maintenance	189	1.00	5.00	3.76	0.81	–1.18	0.177	1.90	0.35
Passing time	189	1.00	5.00	3.89	1.00	–1.17	0.177	1.08	0.35

### Motives for Using Social Media

After combining the 24-items to form the factors identified by Hollenbaugh and Ferris (2014), our results indicated that social media use is mainly driven by the ‘Pass Time’ motive. However, there was also a strong desire to ‘Maintain Relationships’. See Table 1 for the finding for each motive. In comparison, Ferris and Hollenbaugh (2018) reported the following results for ‘Virtual Community’ (*M* = 2.02, *SD* = 0.92, α = 0.89), ‘Companionship’ (*M* = 1.93, *SD* = 1.07, α = 0.94), ‘Exhibitionism’ (*M* = 2.01, *SD* = 1.00, α = 0.90), ‘Relationship Maintenance’ (*M* = 4.18, *SD* = 0.72, α = 0.87) and ‘Passing Time’ (*M* = 4.05, *SD* = 0.92, *r* = 0.66, *p* < 0.001).

### Individual Characteristics and Motives for Using Social Media

In order to assess the relationship between personality traits and motives for the use of social media, scores for the Big Five factors were correlated with scores for each of the five motives (see Table 2). The factors identified through the motivation scale were not normally distributed (*p* < 0.001), therefore non-parametric Spearman rho correlations were employed in the analyses that follow. Power estimates were calculated using G^∗^Power 3.1.9.4 software, based on an α error probability of 0.05, and are reported in Table 2.

**TABLE 2:** Correlating motives with age and Big Five trait scores.

		Virtual community	Companionship	Exhibitionism	Relationship maintenance	Passing time
Neuroticism	Correlation Coefficient	0.14	0.29**	0.21**	–0.04	0.223**
	Sig. (two-tailed)	0.05	0.00	0.004	0.61	0.002
	Power	0.49	0.98	0.83	0.08	0.87
	*N*	189	189	189	189	189
Extraversion	Correlation coefficient	–0.04	–0.13	0.10	0.20**	0.02
	Sig. (two-tailed)	0.58	0.080	0.17	0.01	0.78
	Power	–0.04	0.43	0.28	0.79	0.06
	*N*	189	189	189	189	189
Openness	Correlation coefficient	–0.02	0.09	0.09	0.18*	0.24**
	Sig. (two-tailed)	0.77	0.23	0.21	0.01	0.001
	Power	0.06	0.23	0.23	0.70	0.92
	*N*	189	189	189	189	189
Agreeableness	Correlation coefficient	–0.14	–0.05	–0.27**	0.13	0.002
	Sig. (two-tailed)	0.05	0.52	0.00	0.08	0.98
	Power	0.49	0.10	0.97	0.43	0.05
	*N*	189	189	189	189	189
Conscientiousness	Correlation coefficient	–0.24**	–0.25**	–0.24**	0.15*	–0.19*
	Sig. (two-tailed)	0.001	0.001	0.001	0.04	0.008
	Power	0.92	0.94	0.92	0.54	0.75
	*N*	189	189	189	189	189
Age	Correlation coefficient	–0.18*	–0.29**	–0.20**	0.07	–0.39**
	Sig. (two-tailed)	0.016	0.00	0.01	0.36	0.00
	Power	0.70	0.98	0.79	0.15	0.99
	*N*	189	189	189	189	189

**Correlation is significant at the 0.01 level (two-tailed). *Correlation is significant at the 0.05 level (two-tailed).

Our findings showed a number of moderate relationships between personality and motives for using social media (see Table 2). First, all personality traits except neuroticism were positively related with use of social media to ‘Maintain Relationships,’ however, these relationships were only significant for those scoring higher for extraversion, those scoring higher for openness, and those scoring higher for conscientiousness. In contrast, a small positive correlation was identified between neuroticism and all factors except ‘Relationship Maintenance.’ These positive correlations were significant for all relationships except between neuroticism and ‘Virtual Community.’ Among the many variations between traits, these findings showed particularly clear differences in the motives of individuals scoring higher for neuroticism and individuals scoring higher in conscientiousness with correlations in opposite directions: neuroticism related positively to ‘Companionship,’ ‘Exhibitionism,’ and ‘Passing Time,’ whereas conscientiousness related negatively to the same motives.

Other findings included small but significant negative correlations between conscientiousness and all factors except ‘Relationship Maintenance’ (for which a positive correlation was found), small but significant positive correlations between openness and both ‘Relationship Maintenance’ and ‘Passing Time,’ and a small negative correlation between agreeableness and ‘Exhibitionism.’ No significant relationships were identified between other traits and motives.

As shown in Table 2, age was also significantly negatively correlated with all motives except ‘Relationship Maintenance’ (for which no relationship was found), showing that these are more likely to be recognized as motives by younger participants. To investigate differences between age groups, we split our sample into four groups according to the generation they belonged to (according to Pew Research Center, 2019), from Baby Boomers to Generation Z. This data is displayed in Tables 3, 4. The Wilks’ Lambda value for the differences between generations was 0.697 with a significance value of <0.001. Mean generational differences in the motives for social media use were found to be significant for the ‘Companionship’ and ‘Passing Time’ motives, although allowing for a Bonferroni adjustment only the generational differences for the ‘Passing Time’ motive was significant.

**TABLE 3:** Descriptive statistics for generational motives.

	Generation	Mean	Standard deviation	*N*
Virtual community	Silent (75–92)	1.25	0.35	2
	Boomers (56–74)	1.91	0.89	20
	Generation X (40–55)	1.85	0.92	38
	Millennials (24–39)	1.85	0.80	101
	Generation Z (8–23)	2.26	0.99	28
	Total	1.91	0.87	189
Companionship	Silent (75–92)	2.00	1.41	2
	Boomers (56–74)	2.19	1.12	20
	Generation X (40–55)	2.19	1.13	38
	Millennials (24–39)	2.60	1.08	101
	Generation Z (8–23)	3.04	0.92	28
	Total	2.54	1.10	189
Exhibitionism	Silent (75–92)	1.00	0.00	2
	Boomers (56–74)	1.58	0.68	20
	Generation X (40–55)	1.62	0.76	38
	Millennials (24–39)	1.76	0.75	101
	Generation Z (8–23)	1.84	0.75	28
	Total	1.72	0.74	189
Relationship maintenance	Silent (75–92)	3.60	0.28	2
	Boomers (56–74)	3.65	1.01	20
	Generation X (40–55)	3.84	0.91	38
	Millennials (24–39)	3.75	0.75	101
	Generation Z (8–23)	3.79	0.75	28
	Total	3.76	0.81	189
Passing time	Silent (75–92)	3.00	1.41	2
	Boomers (56–74)	2.80	1.24	20
	Generation X (40–55)	3.49	1.05	38
	Millennials (24–39)	4.20	0.79	101
	Generation Z (8–23)	4.20	0.70	28
	Total	3.89	1.01	189

**TABLE 4:** Tests of between-subjects effects for generational motives.

Dependent variable	Type III sum of squares	df	Mean square	*F*	Sig.	Partial eta squared
Virtual community	4.78	4	1.20	1.61	0.17	0.03
Companionship	15.05	4	3.76	3.24	0.01	0.07
Exhibitionism	2.43	4	0.61	1.10	0.36	0.02
Relationship maintenance	0.54	4	0.14	0.20	0.94	0.004
Passing time	43.73	4	10.93	13.62	0.00	0.23

Five hierarchical multiple regressions were then conducted to determine whether personality traits significantly predicted each of the five motives for social media use, controlling for age and gender (see Table 5). Age and gender were first added to the model, followed by all five-factor personality traits. Firstly, our findings showed that age was a significant negative predictor of the motives to use social media for ‘Companionship,’ ‘Exhibitionism,’ and ‘Passing Time.’ As age increases, these motives decreased. Gender was also found to be a predictor of two motives; females were more likely than males to identify ‘Relationship Maintenance’ and ‘Passing Time’ as motives for social media use.

**TABLE 5:** Regressing motives on individual characteristics.

Predictors	Virtual community			Companionship			Exhibitionism			Relationship maintenance			Passing time		
					
Step 1	β	*t*	Sig.	β	*t*	Sig.	β	*t*	Sig.	β	*t*	Sig.	β	*t*	Sig.
Age	–0.14	–1.90	0.06	−0.25**	–3.47	0.001	−0.20**	–2.70	0.01	0.04	0.53	0.60	−0.43***	–6.68	0.00
Gender	0.004	0.05	0.96	0.06	0.78	0.44	–0.01	–0.18	0.86	0.23**	3.22	0.002	0.18**	2.82	0.01
*R*^2^	0.02			0.07			0.04			0.05			0.25		
*F*	1.89			7.09***			3.71*			5.19**			31.08***		
**Step 2**															
Age	–0.05	–0.64	0.52	−0.16*	–2.09	0.04	–0.05	–0.68	0.50	0.07	0.91	0.36	−0.36***	–5.10	0.00
Gender	0.55	0.70	0.49	0.04	0.55	0.59	–0.03	–0.34	0.73	0.15	1.90	0.06	0.16*	2.25	0.03
Neuroticism	0.03	0.25	0.81	0.19	1.71	0.09	0.29**	2.68	0.01	0.20	1.76	0.08	0.13	1.25	0.22
Extraversion	0.10	1.02	0.31	–0.001	–0.01	0.99	0.30**	3.19	0.002	0.21*	2.12	0.04	0.05	0.60	0.55
Openness	–0.03	–0.37	0.71	0.06	0.75	0.45	0.03	0.40	0.69	0.11	1.44	0.15	0.12	1.77	0.08
Agreeableness	–0.09	–1.16	0.25	0.01	0.14	0.89	−0.16*	–2.11	0.04	0.04	0.53	0.60	0.01	0.15	0.88
Conscientiousness	−0.24*	–2.52	0.01	–0.09	–0.91	0.36	–0.09	–0.97	0.33	0.14	1.44	0.15	–0.06	–0.70	0.48
*R*^2^	0.09			0.13			0.17			0.11			0.28		
*R*^2^ change	0.07			0.06			0.13			0.05			0.03		
F change	2.75*			2.38*			5.47***			2.13			1.64		

All betas are standardized betas. *N* = 189. ***Correlation is significant at the 0.001 level. **Correlation is significant at the 0.01 level. *Correlation is significant at the 0.05 level.

Our findings then showed that models including personality traits improved the explanation of variance for all motives. The *R*^2^ value change for each model demonstrated that five-factor personality traits increased the explanation of variance beyond age and gender alone. The overall explanation of variance was strongest for the prediction of ‘Passing Time’ as a motive, although the F change indicated that the change following the addition of personality traits was not statistically significant. The explanation of variance was weakest for the prediction of ‘Virtual Community’ as a motive. None of the models explained more than 28 per cent of the variance, signaling that other variables explained the majority of the variance in these models.

The improvement in the *R*^2^ value was greatest for the ‘Exhibitionism’ motive, indicating that five-factor personality traits contribute more to this model than other motives. Yet only a few individual personality traits contributed significantly to these models. These included extraversion predicting ‘Relationship Maintenance,’ and neuroticism predicting ‘Exhibitionism,’ both supporting the relationships previously identified through correlations. Other findings included extraversion predicting ‘Exhibitionism,’ agreeableness negatively predicting ‘Exhibitionism,’ and conscientiousness negatively predicting ‘Virtual Community.’ No significant predictive relationships were identified between other traits and motives.

### Duration of Social Media Use

**FIGURE 1:**
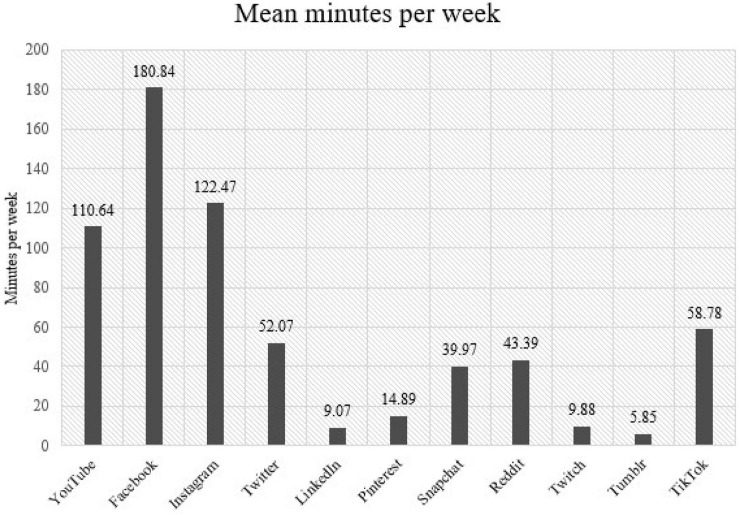
Time spent using each platform per week.

Because duration data were only available from participants owning Apple iPhones, we were able to collect duration data from a subset (*n* = 78) of the total participants (*n* = 189). However, this data indicated the most heavily used social media. Figure 1 displays the time that participants spent using each platform on their iPhones in minutes per week. Our full findings (Table 6) demonstrated that Facebook is the most heavily used platform, with users spending approximately 3 h per week on Facebook on average. Instagram was the second most-used platform, although participants spent almost an hour less on Instagram per week. In comparison, participants spent just 9 min on each of LinkedIn and Tumblr (*M* = 5.86 min).

**TABLE 6:** Descriptive statistics for duration of social media use.

	*N*	Range	Minimum	Maximum	Mean	Standard deviation
YouTube (mins)	78	1225.00	0.00	1225.00	119.24	225.81
Facebook (mins)	78	2591.00	0.00	2591.00	181.74	340.30
Instagram (mins)	78	1024.00	0.00	1024.00	125.49	185.75
Twitter (mins)	78	441.00	0.00	441.00	52.08	103.83
LinkedIn (mins)	78	248.00	0.00	248.00	9.82	32.61
Pinterest (mins)	78	304.00	0.00	304.00	16.23	48.78
Snapchat (mins)	78	600.00	0.00	600.00	40.92	105.32
Reddit (mins)	78	1488.00	0.00	1488.00	42.06	187.00
Twitch (mins)	78	303.00	0.00	303.00	10.46	42.23
Tumblr (mins)	78	180.00	0.00	180.00	9.68	31.53

Motives, age, and five-factor personality traits were also correlated with the duration data for each platform using Spearman rho correlations. Our results (see Table 7) showed statistical significance for the correlations between both the ‘Companionship’ and ‘Passing Time’ motives and the duration of Instagram and Snapchat use. A correlation was also identified between the ‘Passing Time’ motive and duration of Twitter use. The ‘Relationship Maintenance’ motive correlated with the duration of use of Facebook. Neuroticism correlated significantly and positively with Instagram, Twitter and Reddit; yet extraversion correlated negatively with Reddit. Age correlated negatively and significantly with Instagram, Twitter and Snapchat, showing that duration of use for these platforms was higher among those who were younger. Again, we split our sample into generational groups; however, the Wilks’ Lambda value for the differences between generations was 0.578 with a significance value of 0.142, indicating that these differences were not significant.

**TABLE 7:** Correlating duration of use, with motives, personality traits, and age.

	YouTube (mins)	Facebook (mins)	Instagram (mins)	Twitter (mins)	LinkedIn (mins)	Pinterest (mins)	Snapchat (mins)	Reddit (mins)	Twitch (mins)	Tumblr (mins)
Virtual community	0.12	–0.02	0.22	0.12	0.05	0.10	0.09	0.14	0.12	0.09
Companionship	0.04	0.14	0.26*	0.16	–0.02	0.07	0.27*	0.12	0.03	0.02
Exhibitionism	0.06	0.06	0.18	–0.08	–0.05	–0.001	0.12	–0.06	–0.05	–0.03
Relationship maintenance	0.07	0.28*	–0.03	0.03	0.03	0.12	0.11	–0.10	–0.01	0.01
Passing time	–0.05	0.05	0.33**	0.23**	–0.09	0.02	0.25*	0.06	0.03	–0.02
Neuroticism	0.17	0.09	0.28*	0.30*	0.01	0.02	0.19	0.25*	0.14	0.20
Extraversion	–0.10	0.06	–0.06	–0.22	–0.03	0.05	0.13	−0.26*	–0.13	–0.18
Openness	–0.13	–0.01	0.13	0.03	–0.03	0.09	–0.16	–0.01	–0.09	–0.02
Agreeableness	–0.003	0.15	–0.01	–0.01	0.05	0.12	–0.15	–0.17	–0.09	–0.10
Conscientiousness	–0.14	–0.01	–0.18	–0.15	0.07	0.14	0.03	–0.17	0.08	0.04
Age	–0.13	0.07	−0.38**	−0.32**	0.05	0.03	−0.48**	–0.001	–0.09	–0.07

**Correlation is significant at the 0.01 level (two-tailed). *Correlation is significant at the 0.05 level (two-tailed).

## Discussion

This exploratory study has identified a number of findings about social media use in situations requiring physical distancing, including individuals’ most prominent motives for using social media, and the differences in motives for social media use according to personality traits. The previous study to use this scale (Ferris and Hollenbaugh, 2018) assessed motives when participants were not living under physically distanced conditions. As with this previous study, ‘Passing Time’ and ‘Relationship Maintenance’ remain the main motives of social media use. However, the comparison of the mean score in our study for the ‘Companionship’ motive and the mean score for the same motive in the study undertaken by Ferris and Hollenbaugh (2018) indicates that ‘Companionship’ is now a stronger motive than previously identified. This investigation therefore highlights a difference for this motive in a situation where the importance of social media for social interaction with ‘companions’ is amplified by an absence of physical ‘face-to-face’ interaction.

It is also important to acknowledge the effect of both age and gender on the motives for using social media. Similar to Ferris and Hollenbaugh (2018) findings, age is a highly statistically significant negative predictor of the ‘Passing Time’ motive. However, in our study, age was also a statistically significant negative predictor of the ‘Companionship’ motive. Also, unlike Ferris and Hollenbaugh’s (2018) findings, female participants were more likely to identify the ‘Passing Time’ motive. Due to the effects of age and gender therefore, these were controlled for when investigating the relationships between personality traits and motivation as discussed below.

### Personality and Motivation to Use Social Media

Although descriptive statistics reveal trends in the reasons for social media use, a deeper research objective was to understand how individual characteristics affect these motives. Our findings indicate that under physical distancing conditions, motives for social media use vary according to personality traits, suggesting that social media does not serve individuals’ needs in a uniform way. Personality traits also help to predict the ‘Virtual Community’ motive, ‘Companionship’ motive, and ‘Exhibitionism’ motive, although the overall contribution of personality traits to each model is modest.

However, an examination of the contribution made by specific traits reveals some notable differences in the relationships between specific traits and each motive. Similar studies in non-pandemic environments (Hollenbaugh and Ferris, 2014; Ferris and Hollenbaugh, 2018) provide an important benchmark for comparison, against which our findings indicate some clear changes. For example, surprisingly, Ferris and Hollenbaugh (2018) identified no statistically significant effect of extraversion on any motives to use social media. Yet our study found a small but statistically significant positive effect on the ‘Exhibitionism’ and ‘Relationship Maintenance’ motives. Our study therefore suggests that in conditions of enforced physical distancing, those scoring higher for extraversion are more motivated to use social media for ‘Exhibitionism’ and ‘Relationship Maintenance.’ Importantly, in the context of this scale, ‘Relationship Maintenance’ refers to the process of interacting with existing friends, rather than building a new ‘Virtual Community’ or seeking ‘Companionship’ to prevent loneliness (Hollenbaugh and Ferris, 2014). In a physically distanced situation, the finding that those scoring highly for extraversion now use social media for ‘Relationship Maintenance’ suggests that social media use may replace ‘face-to-face’ interaction with these existing friends. These findings also reflect offline studies that have previously related extraversion to being ‘gregarious’ (Costa and McCrae, 1992) and sociable (Argyle and Lu, 1990; Eysenck et al., 1992; Olson and Weber, 2004), regularly conversing with others (Mehl et al., 2006) and engaging in non-verbal communication (Akert and Panter, 1988).

Although not previously identified as a specific motive (Ferris and Hollenbaugh, 2018), some aspects of online relationship-maintaining behavior have previously been related to extraversion. Gregarious and sociable tendencies have previously been revealed on social media via indicators such as high friend quantity (see Amichai-Hamburger and Vinitzky, 2010; Gosling et al., 2011; Ong et al., 2011) and interaction within groups (Bachrach et al., 2012; Kelsen and Flowers, 2018). Furthermore, studies have previously identified a desire to ‘connect’ on social media (Bhattacharya et al., 2014; Orchard et al., 2014). However, our novel findings suggest that under social distancing conditions this social online behavior is now an explicit reason for social media use.

In their recent study, Ferris and Hollenbaugh (2018), identified that neuroticism had a small but significant positive effect on the ‘Companionship’ motive. Although our findings reveal a relationship in the same direction, it is not statistically significant. Furthermore, we find a small statistically significant effect for neuroticism on the ‘Exhibitionism’ motive. This is a surprising finding, given that those with high trait neuroticism are typically socially uneasy (de Jong et al., 1999; Tong et al., 2004; Dehle and Landers, 2005; Suurmeijer et al., 2005). The contrast with the finding within the previous study (Ferris and Hollenbaugh, 2018) therefore suggests that ‘Exhibitionism’ is specifically a motive under physically distanced conditions. Some of this apparent intention to self-promote might be explained by the self-consciousness facet contributing to neuroticism (Costa and McCrae, 1992) within the five-factor model. Previous studies have also revealed a positive relationship with use of social networks for self-promotion (Roulin, 2014), and ‘commenting’ as a form of ‘exhibitionism’ (Wu and Atkin, 2017). Our finding suggests that these may become more prominent motives under physically distanced conditions.

The previous study (Ferris and Hollenbaugh, 2018) reported that openness had a small but significant positive effect on the ‘Companionship,’ ‘Exhibitionism,’ and ‘Relationship Maintenance’ motives. Although we find similar effects, none of these was statistically significant. Although the lack of a significant relationship might initially appear to contradict the relationship between openness and use of social media to connect with like-minded people (Bhattacharya et al., 2014), prior research into social media use among those high in openness is contradictory anyway. While some studies indicate that those who use social media are likely to score higher for openness (Özgüven and Mucan, 2013; Buettner, 2016; Taber and Whittaker, 2018), others have found a negative correlation between social media use and openness (Annisette and Lafreniere, 2017). Therefore, drivers of social media use are one aspect of the relationship between social media and openness requiring further attention in order to understand these inconsistencies.

Ferris and Hollenbaugh (2018) reported that agreeableness had a small but significant positive effect on the ‘Virtual Community’ motive and small but significant negative effect on the ‘Exhibitionism’ motive. Although not statistically significant, our study now finds a negative relationship with the ‘Virtual Community’ motive. This is a surprising finding requiring further investigation, especially since other studies have shown that agreeableness does relate to use of social media for social interaction (Eşkisu et al., 2017), and that people higher in agreeableness are driven by the potential for social benefit (Marino et al., 2016). However, reflecting the previous study (Ferris and Hollenbaugh, 2018), we also find a small but significant negative effect on the ‘Exhibitionism’ motive. This again reflects the ‘modesty’ of agreeable people (Costa and McCrae, 1995) who consistently feel uncomfortable ‘showing off’, as reflected previously in the negative relationship with self-status seeking on social media (Lin et al., 2017).

In Ferris and Hollenbaugh’s (2018) study, conscientiousness had a small but significant negative effect on the ‘Virtual Community,’ ‘Companionship,’ and ‘Exhibitionism’ motives. We also find small negative relationships for each of these motives, although only the effect on the ‘Virtual Community’ motive is statistically significant. These results indicate that conscientious people, who are known to be ‘self-disciplined’ (Costa and McCrae, 1995) and focused on the task in hand, do not allow social media use to distract them. Yet, again there is some contradiction between our findings and wider findings that show that a relationship between conscientiousness and the use of social media to connect with peers and new friends (Bhattacharya et al., 2014), use of Facebook for maintaining relationships Horzum (2016), and use of Twitter for ‘social purposes’ Hughes et al. (2012).

### Duration of Social Media Use and Relationship to Personality Traits

As the world’s most popular platform according to membership (Statista, 2020), it is perhaps unsurprising that the social media platform used for the longest duration is Facebook. Our data also shows that Facebook use is also related to the motive to ‘Maintain Relationships.’ However, few significant relationships were identified between personality traits and duration of social media use. Of the relationships that were identified, three significant correlations were found between neuroticism and use of Instagram, Twitter, and Reddit. Whilst previous studies have identified positive correlations between neuroticism and duration of social media use, these have previously focused on Facebook (Moore and McElroy, 2012; Kuo and Tang, 2014). In fact, previous studies of Instagram (such as Brailovskaia and Margraf, 2018; Casado-Riera and Carbonell, 2018) and Twitter (such as Petrocchi et al., 2015; Yoong et al., 2017) have not identified a link to neuroticism at all.

### Limitations and Future Research Directions

While this study provides an important insight into motivation under physically distanced conditions, our findings are based on a relatively small sample of 189 respondents. This is therefore an exploratory study providing indicative findings and signposting topics for further research. Although significant relationships were identified, there is clearly an opportunity to repeat this research with a wider sample in order to compare results across nations and cultures, as suggested by Henrich et al. (2010). Within a wider sample we also recognize the opportunity to explore the effect of further potential variables such as educational level or employment, neither of which were examined in our study nor the preceding Hollenbaugh and Ferris (2014) study. This sample included a wide range of ages (18–75 years with a large standard deviation), whereas future samples might enable comparison between specific age groups. Our study also indicated two motives for females specifically. This requires further investigation. Firstly, there is an opportunity to understand why these motives are particularly found for females. Secondly, further work is required to understand what the motives are for male social media use and why these are different.

Given predictions about new patterns of behavior emerging as the ‘lasting digital legacy’ of COVID-19 (Ofcom, 2020b), this research suggests wider changes in the motivations for social media use that may be longer-term in a post-pandemic world. Further research could also assess whether the trends found in this specific ‘pandemic’ situation are replicated in other situations where people are reliant on social media to maintain a relationship such as those whose professions take them physically away from social circles. This might include, for example, those in the military, regular long-distance travelers, or those working in remote locations such as miners. Furthermore, a future replication of the study when the pandemic has ended would provide an important comparison point to identify the extent to which these findings are specific to a pandemic environment. Longitudinal studies might also provide insight on longer-term changes in social media use, both resulting from the constant evolution in social media functionality as well as legacy changes to communication practices following the pandemic.

Our study employed the 120-item IPIP-NEO-120 scale provided by Johnson (2014), whereas the previous study against which we compare our results used the 44-item Big Five Inventory (BFI) scale provided by John et al. (1991), so we acknowledge that each measurement of the big five traits used a slightly different set of questions. Yet, previous comparisons of IPIP and BFI items (such as Donnellan et al., 2006; Zheng et al., 2008; Fossati et al., 2011; Akhtar and Azwar, 2018) have indicated that there is a strong correlation between the measures. We also recognize the subtle difference between our study which investigated motives for ‘social media’ use in general, and our comparison with a previous study which focused on motives for one specific social media platform, Facebook. Our duration data did demonstrate that Facebook was the most-used platform, indicating that despite the question referring to ‘social media,’ participants were likely to be the describing motives for Facebook use. Nevertheless, a future study might more clearly compare motives for ‘social media’ between physically distanced and non-physically distanced situations. This might involve comparison of two samples, or one sample during and post-pandemic, using a *t*-test. Comparison of samples was not possible in the current study as the previous data was not available. There is also an opportunity to apply the same measures across each of the commonly used social media platforms in turn to study nuances in the motives associated with each. For example, is the motivation to use a messaging platform designed specifically to engage the user in ‘active’ use different to that of a video platform primarily intended to broadcast to a largely ‘passive’ audience?

Lastly, there is a limitation with our duration data as we were only able to collect this from Apple iPhone users. We acknowledge that this data therefore does not represent the full sample, plus we recognize the possibility that the characteristics of Apple iPhone users may affect their usage of social media. Therefore, a consistent methodology is required to collect accurate social media duration data from users of all technologies, including those who access social media using computers.

In conclusion therefore, based on a novel situation, this study has identified compelling data about the motives to use social media, how these relate to individual characteristics such as personality traits, and how the data compares to similar pre-pandemic data. However, through further research this could lead to a fuller and more accurate picture about the conditions that might lead to similar findings. Given the doubts raised about when or even whether society will ever return to the same frequency of physical interaction, this is an intriguing and potentially important area for future exploration.

## Data Availability Statement

The raw data supporting the conclusions of this article will be made available by the authors, without undue reservation.

## Ethics Statement

The studies involving human participants were reviewed and approved by the University of Bath. Written informed consent for participation was not required for this study in accordance with the national legislation and the institutional requirements.

## Author Contributions

TB-G undertook the research and wrote the manuscript, with supervision, review, and editing by JH and AJ. All authors contributed to the article and approved the submitted version.

## Conflict of Interest

The authors declare that the research was conducted in the absence of any commercial or financial relationships that could be construed as a potential conflict of interest.

## Footnotes

1 www.prolific.co
